# Analysis of the Reasons for Poor Prognosis in Severe to Profound Sudden Sensorineural Hearing Loss: A Systematic Review and Meta-Analysis

**DOI:** 10.3390/diagnostics15212770

**Published:** 2025-10-31

**Authors:** Linrui Chen, Jianhui Qiu, Qianqian Zhang, Zian Xi, Qiong Wu, Mingwei Xu, Qin Zhang, Yulian Jin, Jun Yang, Maoli Duan, Qing Zhang, Zhiyuan Zhang

**Affiliations:** 1 Department of Otorhinolaryngology Head and Neck Surgery, The First Affiliated Hospital of Nanchang University, Nanchang 330006, China; lrc_study@163.com (L.C.); jianhuiqiu0313@163.com (J.Q.); 2 Department of Otolaryngology Head and Neck Surgery, Xinhua Hospital, Shanghai Jiaotong University School of Medicine, Shanghai 200092, China; entdr_qian@163.com (Q.Z.); wq19980518@163.com (Q.W.); mw.xu@outlook.com (M.X.); zhangqin5235@126.com (Q.Z.); jinyulian8548@xinhuamed.com.cn (Y.J.); yangjun@xinhuamed.com.cn (J.Y.); 3 Ear Institute, Shanghai Jiaotong University School of Medicine, Shanghai 200092, China; 4 Shanghai Key Laboratory of Translational Medicine in Ear and Nose Diseases, Shanghai 200092, China; 5 Department of Otolaryngology-Head and Neck Surgery, Sir Run Run Shaw Hospital, Medical College of Zhejiang University, Hangzhou 310016, China; 6 International Medical School, Chongqing Medical University, Chongqing 400016, China; 18279148241@163.com; 7 Division of Ear, Nose and Throat Diseases, Department of Clinical Science, Intervention and Technology, Karolinska Institute, SE-171 77 Stockholm, Sweden; 8 Ear Nose and Throat Patient Area, Trauma and Reparative Medicine Theme, Karolinska University Hospital, SE-171 76 Stockholm, Sweden

**Keywords:** sudden sensorineural hearing loss, meta-analysis, prognosis, severe to profound, risk factors

## Abstract

**Objectives:** Patients with severe to profound sudden sensorineural hearing loss (SSNHL) generally experience poorer hearing recovery; however, the associated risk factors have not been identified. This study synthesizes current evidence to explore prognostic risk factors in this patient group. **Methods:** Databases were systematically searched through PubMed, Embase, Web of Science, and the Cochrane Library, from their inception to 18 October 2025. Three researchers independently extracted and recorded patient information and relevant data from all selected studies. Any inconsistencies were clarified through discussion or by consulting a fourth researcher. **Results:** The study included 2632 patients from 15 articles published between 2002 and 2025 and evaluated 8 prognostic risk factors. The results showed that profound hearing loss (OR = 4.68; 95% CI: 3.57–6.13; *p* < 0.001) and vertigo (OR = 1.95; 95% CI: 1.28–2.98; *p* = 0.002) were correlated with poorer hearing recovery. Subgroup analyses based on different prognostic criteria confirmed the consistent impact of hearing loss severity on poor outcomes. The remaining 6 risk factors did not show statistically meaningful associations. **Conclusions:** Profound hearing loss and vertigo are significantly associated with poorer prognosis in patients with severe to profound SSNHL. These findings may help identify high-risk patients early and inform the design of personalized therapeutic approaches in clinical settings.

## 1. Introduction

Sudden sensorineural hearing loss (SSNHL) is considered an otologic emergency and clinically defined as a sudden decline in hearing of ≥30 dB HL in at least three contiguous frequencies within 72 h [1]. SSNHL usually occurs unilaterally and is often accompanied by additional symptoms, such as vertigo, tinnitus, and aural fullness. In recent years, there has been a growing global incidence of SSNHL, and its treatment and prognosis have become a hot topic in current research. Epidemiological surveys in the United States have shown that the annual incidence of SSNHL is approximately 5–27 cases per 100,000 people [2]. In Germany, the incidence of SSNHL is 160 cases per 100,000 inhabitants [3] or even up to 400 cases [4]. In Japan, the number of patients seeking medical attention for SSNHL has significantly increased, rising from 4000 cases in 1972 to 35,000 cases in 2001 [5]. However, since some SSNHL patients may recover spontaneously, the actual incidence may be even higher [6]. The etiology of SSNHL may be associated with viral infections, vascular disorders, autoimmune diseases, tumors, inflammation, and so on [6], and overall treatment outcomes are often unsatisfactory.

Current therapeutic guidelines recommend systemic corticosteroids as first-line treatment, potentially combined with adjuvant modalities including hyperbaric oxygen therapy, antiviral agents, and vasoactive compounds [1,6]. Although various factors have been proposed as prognostic factors of the disease, including initial hearing loss, vertigo, comorbidities such as diabetes and hypertension, and the time window from onset to treatment [7,8], the conclusions are still controversial. While some patients experience hearing improvement through treatment or spontaneous recovery, a considerable proportion have poor outcomes, particularly those with severe to profound hearing loss, whose recovery rates are much lower than those with mild to moderate hearing loss [9]. These patients often present no significant hearing improvement or even permanent hearing impairment, leading to long-term challenges such as communication barriers, social isolation, and psychological issues [10], which have become an important challenge in the field of otology.

Although several meta-analyses have explored prognostic factors for SSNHL, most of their inclusion criteria have not been stratified for the subgroup of severe to profound hearing loss [11,12]. Notably, this subgroup exhibits the highest disability rates and represents a major challenge in the clinical management of SSNHL. Therefore, we analyzed existing evidence to investigate the relationship between clinical characteristics and poor prognosis in patients with severe to profound SSNHL, aiming to identify key factors influencing hearing recovery and to provide more clinically relevant evidence. This will enhance the understanding of severe to profound SSNHL and provide a theoretical basis for the clinical practice, ultimately improving patients’ hearing recovery and life satisfaction.

## 2. Materials and Methods

The literature identification and selection process was carried out following the PRISMA 2020 (Preferred Reporting Items for Systematic Reviews and Meta-Analyses) guidelines [13], and the protocol was registered in the International Prospective Register of Systematic Reviews (PROSPERO) under the registration number CRD420251171241. Three researchers (LRC, JHQ, and QQZ) independently completed all steps, and a fourth researcher (QZ) resolved any discrepancies in the results.

### 2.1. Search Strategy

This study used the Population, Intervention, Comparison, Outcome, and Study (PICOS) question to systematically search the literature:(P) patients with severe to profound SSNHL (I) treated with steroids and other treatments (C) good prognosis (O) poor prognosis (S) prospective or retrospective studies. The database search covered PubMed, Embase, Web of Science, and the Cochrane Library from the establishment of the database to 18 October 2025. The search strategy combined the medical subject heading (MeSH) terms with free-text words: “hearing Loss, sudden” [MeSH], “sudden hearing loss”, “deafness, sudden”, “sudden deafness”, “idiopathic sudden sensorineural hearing loss “, “sudden sensorineural hearing loss”, “SSNHL”, “SNHL”, “severe to profound”, “severe-to-profound”, “severe”, “profound”, “prognosis” [MeSH], “prognoses”, “prognostic factors”, “prognostic factor”, “factor, prognostic”, “factors, prognostic”, “age”, “vertigo”, “tinnitus”, “hypertension”, “diabetes”. Additionally, we manually reviewed the reference lists of the literature to capture any relevant studies not retrieved through database searches. All retrieved articles were uploaded to the literature management software EndNote 21 (Clarivate; Philadelphia, PA, USA) for screening.

### 2.2. Criteria for Study Selection

Inclusion criteria:

(1) Studies provide relevant data on patients with severe to profound SSNHL;
(2) Prospective or retrospective studies comparing the prognosis of SSNHL;
(3) Studies with clearly defined efficacy evaluation criteria;
(4) Studies provide or allow the calculation of odds ratios (ORs) and 95% confidence intervals (CIs).

Exclusion criteria

(1) Studies with inaccessible full text despite retrieval attempts;
(2) Studies with small sample sizes (<30 cases) or low quality (NOS < 5), as small-sample studies tend to produce unstable or biased effect estimates [14];
(3) Basic research or animal experiments.;
(4) Reviews, case reports, or conference abstracts.

### 2.3. Data Extraction

Data extraction was conducted independently by two researchers (MWX and QZ). Any disagreements were resolved through discussion. The extracted data included: the first author, country of study, study design, sample size of patients, treatment regimen, hearing improvement criteria, and prognostic factors. Differences in the definitions of SSNHL and the criteria for the grading of hearing loss across studies were also recorded.

### 2.4. Hearing Loss and Prognostic Criteria

Severe to profound hearing loss was defined as an initial pure tone average (PTA) ≥ 60 dB HL. According to the Siegel criteria [15], a poor prognosis was defined as a hearing gain of less than 15 dB HL combined with a final PTA exceeding 75 dB HL (Table 1).

### 2.5. Quality Assessment

We used the Newcastle-Ottawa Scale (NOS) [16] for the quality assessment of the included cohort studies and the Joanna Briggs Institute (JBI) [17] critical appraisal tool for the quality assessment of the included case series studies. The NOS rates studies in three domains, with a maximum of 9 points. Scores ≥ 7 indicate high quality, 5–6 moderate, and <5 low quality. The JBI critical appraisal tool includes 10 questions, with a total score of 10 points, and studies scoring ≥ 5 points were considered high quality. Two researchers (ZAX and QW) completed the quality assessment independently, and any disagreements were resolved through discussion or adjudication by a third researcher (QZ). The specific quality scores for each study are listed in Tables S1 and S2.

### 2.6. Data Analysis

Meta-analysis was performed using Cochrane Review Manager (RevMan) version 5.4 (The Cochrane Collaboration 2020, London, UK). Effect size synthesis: Odds ratios (ORs) and 95% confidence intervals (95% CIs) were calculated or extracted and combined using the Generic Inverse Variance method. Assessment of heterogeneity: Heterogeneity among studies was evaluated using the chi-squared (χ^2^) test combined with the I^2^ statistic. If *p* > 0.10 and I^2^ < 50%, it indicates statistical homogeneity among studies, and a fixed-effects model was used for the meta-analysis. If *p* ≤ 0.10 and I^2^ ≥ 50%, it suggests significant heterogeneity among studies, and a random-effects model was applied. Clinically, this heterogeneity suggests differences in patient characteristics, diagnostic criteria, or treatment protocols across studies; therefore, pooled estimates should be interpreted with caution when applied to specific clinical settings. Sensitivity analysis was performed to explore the sources of heterogeneity by sequentially excluding individual studies and observing changes in the pooled effect size to assess the stability of the results. The Z-test was used to determine the statistical significance of the pooled results. A *p*-value < 0.05 was considered statistically significant.

## 3. Results

### 3.1. Literature Search

**Figure 1:**
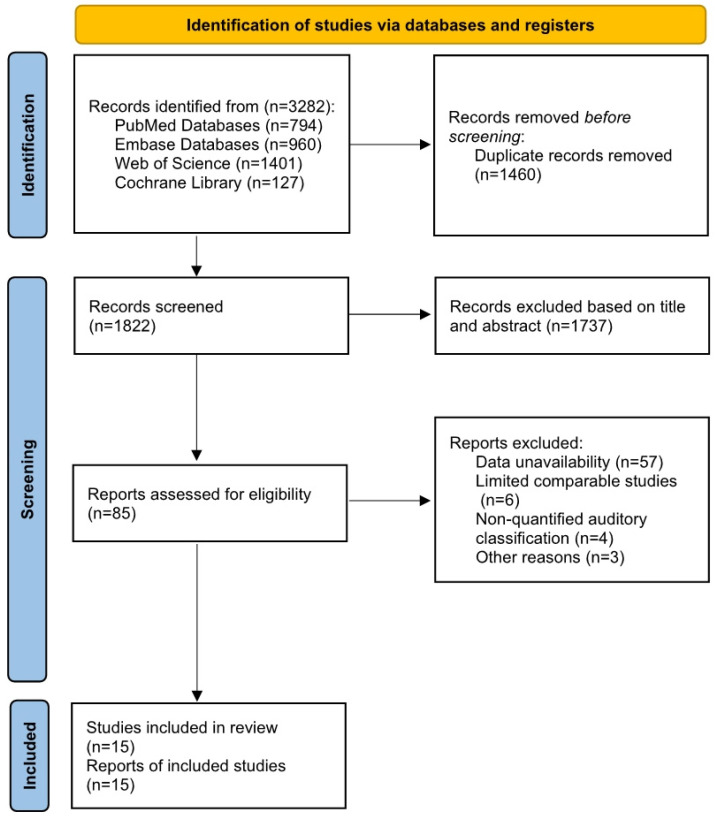
PRISMA flowchart of the systematic literature search process.

A total of 3282 studies were identified through databases in the systematic review. (PubMed: 794, Embase: 960, Web of Science: 1401, Cochrane: 127). After removing 1460 duplicate articles, 1822 studies remained. Following title and abstract screening, studies involving non-human experiments, reviews, and case reports were excluded, leaving 85 studies. In a full-text review of the remaining studies, we excluded 57 studies that failed to extract valid data required for this paper, 6 isolated studies (involving factors such as serum bilirubin levels and thyroid-related hormone levels) due to a lack of support from comparable studies and insufficient evidence, 4 studies without specific values for hearing classification, 1 studies with insufficient age span (not aligned with the purpose of this study), and 2 studies with inaccessible full text despite retrieval attempts. Thus, 15 studies [18,19,20,21,22,23,24,25,26,27,28,29,30,31,32] were included in the final data extraction and analysis. Figure 1 shows the PRISMA flow diagram summarizing the complete literature search process.

### 3.2. Included Study Characteristics

Table 2 summarises the main clinical characteristics of the included studies. The included studies were published between 2002 and 2025, and comprised 15 studies conducted in China (7), South Korea (5), India (1), Brazil (1), and Germany (1), with a total of 2632 patients. The patients’ age ranged from 5 to 93 years. All studies were retrospective, including 7 retrospective case series and 8 retrospective cohort studies. 8 risk factors for poor prognosis were screened, including 2 general clinical characteristics (gender, age > 60 years), profound hearing loss, delayed treatment, 2 accompanying symptoms (tinnitus, vertigo), and 2 comorbidities (hypertension, diabetes). Most studies defined SSNHL as hearing loss of more than 30 dB HL in at least three consecutive frequencies within 72 h [18,19,21,22,23,24,26,27,29,30,32], while 2 study defined it as hearing loss ≥ 20 dB HL in at least two consecutive frequencies within 72 h [28,31], and 2 studies provided definitions specifically for severe or profound SSNHL [20,25]. Different hearing classification criteria were used for the included studies, and the original classifications were retained during data extraction (Table S3). Regarding the hearing recovery criteria, 9 articles used Siegel’s criteria [15], 3 articles referred to the Chinese Medical Association of Otolaryngology criteria [33], and 2 article referred to the guidelines published by the American Academy of Otolaryngology Head and Neck Surgery Foundation (AAO-HNSF) [6], and 1 study did not specify the source of the assessment criteria and possibly using researcher-defined standards [24]. Although each subgroup meta-analysis included fewer than 10 studies, we still generated funnel plots for subgroups with at least 5 studies to preliminarily explore publication bias, including severity of hearing loss, vertigo, and gender. The distribution of effect sizes appeared symmetrical (Figure S1). We also performed sensitivity analyses on each subgroup, and the results indicated that the findings were generally stable and reliable.

### 3.3. Meta-Analysis Results

**Figure 2:**
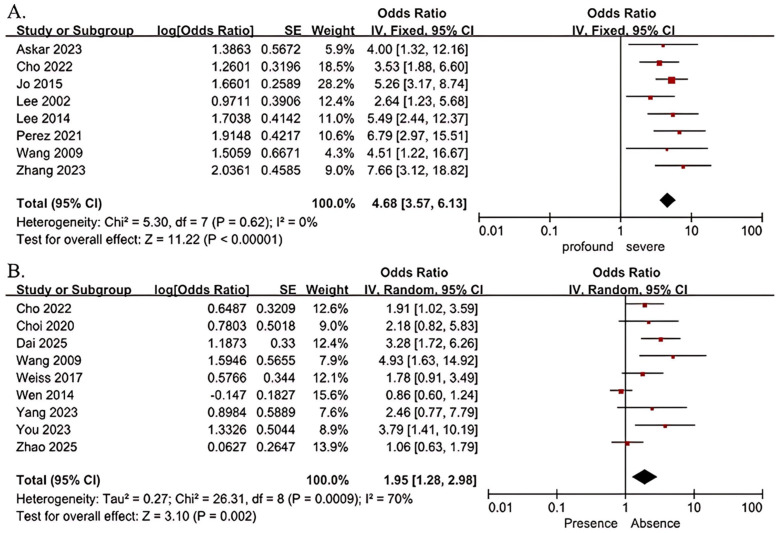
Forest plots of outcomes for subgroups of severity of hearing loss (**A**) [18,19,21,22,23,24,29,30] and vertigo (**B**) [19,20,24,25,26,27,28,31,32]. The right-hand side shows the odds ratio (OR) for each study with 95% confidence interval (CI). Each horizontal line represents the OR and its 95% CI, with line length indicating the CI range. ■ Squares represent the OR of each study, sized according to study weight in the meta-analysis. ◆ The diamond represents the pooled OR, with the center indicating the combined effect and the width representing the 95% CI.

Risk factors for poor clinical prognosis in patients with severe to profound SSNHL were profound hearing loss, gender, age > 60 years, tinnitus, and delayed treatment > 7 days were not statistically heterogeneous between studies (I^2^ < 50%, *p* > 0.10), and were analyzed by meta-analysis using a fixed-effects model; Risk factors for poor clinical prognosis in patients with severe to profound SSNHL for vertigo, hypertension, and diabetes were statistically heterogeneous across studies (I^2^ ≥ 50%, *p* ≤ 0.10), and meta-analyses were performed using a random-effects model. The results showed that profound hearing loss (OR = 4.68; 95% CI 3.57–6.13; *p* < 0.001), and the presence of vertigo (OR = 1.95; 95% CI 1.28–2.98; *p* = 0.002) were risk factors for poor clinical prognosis in patients with severe to profound SSNHL (shown in Figure 2); and that gender, age > 60 years, delayed treatment > 7 days, the presence of tinnitus, hypertension, and diabetes were not risk factors for poor clinical prognosis in patients with severe to profound SSNHL (Table 3).

**Figure 3:**
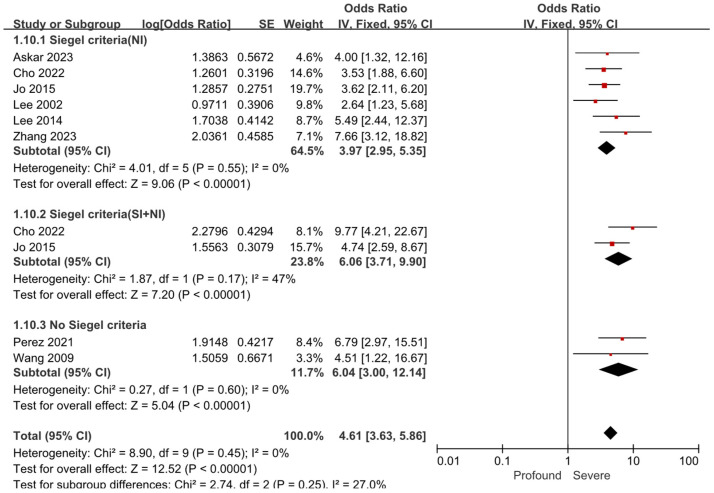
Forest plot of the severity of hearing loss and prognosis by different criteria. Data are grouped by Siegel criteria (NI) [18,19,21,22,29,30], Siegel criteria (SI+NI) [19,21], or No Siegel criteria [23,24]. The right-hand side shows the odds ratio (OR) for each study with 95% confidence interval (CI). Each horizontal line represents the OR and its 95% CI, with line length indicating the CI range. ■ Squares represent the OR of each study, sized according to study weight in the meta-analysis. ◆ The diamond represents the pooled OR, with the center indicating the combined effect and the width representing the 95% CI.

Considering the bias caused by different hearing recovery criteria among the 15 included studies, we conducted a subgroup analysis specifically for the risk factor of the severity of hearing loss. Among the 8 studies that evaluated the association between the severity of hearing loss and prognosis [18,19,21,22,23,24,29,30],6 studies used the Siegel’s criteria, 4 of which [18,22,29,30] defined poor prognosis as a post-treatment hearing gain of less than 15 dB HL, and 2 studies [19,21] reported stratified outcomes, presenting the number of patients in each subgroup, which allowed for recalculation based on the original data. The remaining 2 studies classified prognosis using non-Siegel standards: one study [24] defined poor prognosis as a post-treatment hearing gain of less than 30 dB HL, while another study [23] defined poor prognosis as a post-treatment PTA improvement of less than 10 dB HL or failure to recover to a serviceable hearing level (PTA > 50 dB HL or WRS < 50%). The results demonstrated that regardless of the hearing prognostic criteria used, the pooled results of the impact of the severity of hearing loss on the prognosis of severe to profound SSNHL remained consistent. Compared to patients with severe hearing loss, those with profound loss were at a substantially elevated risk of poor outcomes (OR = 4.61; 95% CI: 3.63–5.86; *p* < 0.001), with the difference remaining statistically significant (shown in Figure 3).

## 4. Discussion

As a common emergency in otology, the prognostic of SSNHL significantly impacts patients’ long-term quality of life. Although SSNHL has a certain tendency for spontaneous recovery, a considerable proportion of patients experience long-term hearing impairment. Therefore, identifying the key prognostic factors is crucial for optimizing clinical diagnosis and treatment strategies. Existing studies have indicated a strong association between the severity of hearing loss and the prognosis of SSNHL. Ceylan et al. [34] found that patients with an initial PTA threshold exceeding 40 dB HL (especially those above 60 dB HL) had poorer recovery rates; some researchers had observed that patients with an initial PTA over 70 dB HL had worse hearing outcomes compared to those with an initial PTA below 70 dB HL [35]; a meta-analysis by Liebau et al. indicated a critical threshold (around 80–90 dB HL) beyond which hearing loss is difficult to improve effectively through natural recovery or therapeutic intervention [36]. These findings suggest that patients with mild to moderate SSNHL have significantly better prognoses compared to those with severe to profound SSNHL. However, there were discrepancies in the criteria for the grading of hearing loss across academic studies. In this study, based on included studies, certain clinical guidelines [37], and the WHO (1997) criteria [38] for defining severe to profound hearing loss, we set the lower inclusion threshold at an initial PTA ≥ 60 dB HL. We focused on the severe to profound SSNHL group (≥60 dB HL) and systematically analyzed their clinical features associated with poor prognosis through a meta-analysis, aiming to provide evidence-based support for early identification of high-risk groups, development of stratified intervention strategies, and optimization of hearing rehabilitation resource allocation.

This systematic review and meta-analysis included 15 studies that investigated or involved clinical features affecting the prognosis of patients with severe to profound SSNHL. By pooling the data, this study systematically explored 8 potential prognostic risk factors. Among them, the severity of hearing loss and the presence of vertigo were found to have statistically significant impacts on patient outcomes, both being significantly associated with an increased risk of poor prognosis, consistent with the conclusions of most studies [39,40,41]. One study found that the overall recovery rate for patients with severe to profound hearing loss was much lower than patients with mild to moderate hearing loss [40]; another study reported that patients with an initial PTA threshold exceeding 100 dB HL had a recovery rate of only 8.1–9.1% [41]. Although there were slight differences in the definition ranges of hearing loss between the two studies, both sets of data showed a consistent pattern: as the initial severity of hearing loss increased, the recovery rate declined. Furthermore, in subgroup analyses using different prognostic criteria, the association between the severity of hearing loss and poor prognosis in patients with severe to profound SSNHL remained significant, making the conclusion even more convincing.

Vertigo is a common accompanying symptom of SSNHL, with an incidence of approximately 30–60%. When the blood supply to the labyrinthine artery is impaired, it can lead to vestibular dysfunction and vertigo. Several studies have demonstrated a higher prevalence of vertigo among patients with profound hearing loss [39,42]. Given the anatomical association between the cochlea and the vestibular system, it can be inferred that vestibular function may be more severely impaired in patients with severe and profound hearing loss. This study found that vertigo is a significant risk factor for poor prognosis in patients with severe to profound SSNHL, consistent with findings from previous research [39,40,43,44,45]. SSNHL patients with vertigo had a significantly lower hearing recovery rate than those without vertigo, suggesting that the presence of vertigo may indicate that the lesion extends to the vestibular system, reflecting broader inner ear damage and subsequently reducing the potential for hearing recovery. Further supporting this, a study by Liu et al. [42] reported that 76.2% of patients with vertigo exhibited abnormal results in 2 to 3 vestibular function tests (cVEMP, oVEMP, and caloric test), whereas patients without vertigo tended to be concentrated in the group with milder damage, indicating a significant association between vertigo and the severity of vestibular end-organ damage.

There was no statistically significant difference between gender or tinnitus and the prognosis of severe to profound sudden SSNHL, consistent with previous studies [7,34]. Tinnitus is the most common complication of SSNHL, occurring in 70–90% of patients, particularly among those with mild to moderate hearing loss. However, the prognostic value of tinnitus in SSNHL remains controversial. Similar to the majority of researchers, this study found that tinnitus does not affect the prognosis of SSNHL. Nevertheless, some scholars have suggested that tinnitus is a risk factor for poor prognosis [46], while others have argued that the presence of tinnitus indicates preserved inner ear hair cell function and may represent a favorable prognostic factor for hearing recovery in SSNHL [39], whereas the absence of tinnitus may imply severe auditory pathway damage or cell death [47]. It is noteworthy that Zou et al. [46] suggested that the presence of tinnitus or its loudness was not significantly associated with prognosis; however, the frequency of tinnitus may have important predictive value for hearing recovery in SSNHL patients.

Age was not significantly associated with prognosis, a finding supported by some studies [34,48]. However, poorer outcomes in elderly patients may be related to increased individual susceptibility, exacerbation of chronic diseases leading to metabolic and inflammatory damage, and age-related declines in hair cell regenerative capacity and neurodegeneration. Thus, most studies have suggested that older age is a risk factor for poorer prognosis [7,43]. However, the critical age thresholds vary among studies, which may be attributed to selection bias in the study populations or significant individual variability among elderly patients, and need to be verified by a larger sample size. The prognosis of SSNHL in children remains controversial. Xiao et al. [49] found that among children with profound SSNHL, the age of those in the effective treatment group (11–12 years) was significantly higher than that of those in the ineffective group (6–9 years). This result is consistent with that of Kim et al. [50]. This may be related to younger children’s limited ability to perceive and express their hearing problems, making it difficult for parents to find unilateral hearing loss early, leading to delayed treatment and, consequently, poorer prognosis. In summary, prognosis in adults is often associated with vascular endothelial dysfunction or metabolic abnormalities, whereas in children, it is more likely to be influenced by indirect factors such as delayed diagnosis and treatment. We speculate that in cases of severe to profound SSNHL, hearing loss is typically caused by serious structural damage to the inner ear, and the irreversibility of such damage may diminish the influence of age-related factors on prognosis. Moreover, these patients often detect the condition earlier due to severe symptoms, thereby reducing age-related differences in delayed treatment and further weakening the direct impact of age on prognosis.

The time window from onset to treatment did not show a statistically significant correlation with prognosis, a finding supported by some studies [51]. However, this finding is controversial compared to the widely accepted view that “early treatment improves prognosis” [34,39]. Ceylan et al. [34] found no correlation between the onset-to-treatment time window and hearing gain, but when the time window was set to 7 days, differences in relative hearing gain and recovery rates were observed. Similarly, Bogaz et al. [39] compared hearing recovery among patients treated within different treatment time windows and found that early treatment significantly improved outcomes, particularly within 7 days. From a pathophysiological perspective, cochlear hair cells have high metabolic activity, making them highly sensitive to ischemia and hypoxia [52]. Studies have shown that after moderate damage (such as ototoxic drugs or noise), inner ear hair cells can regenerate and recover with prompt treatment within a short period; however, if the damage is severe or treatment is delayed, hair cells may suffer irreversible functional loss [53], and even correcting ischemic or hypoxic conditions may not fully restore function. The discrepancies between the findings of this study and those supporting early treatment may be attributed to several factors: first, there was heterogeneity among included studies regarding the duration of delayed treatment and prognosis evaluation criteria, making direct comparison and pooling of results challenging, and there are few subgroups of studies (*n* = 2); second, patients with severe to profound SSNHL had more severe hearing loss compared to other subgroups, and the proportion of patients with delayed treatment was relatively small, potentially limiting the observable effect of treatment delay on prognosis; third, from a pathological perspective, patients with severe to profound SSNHL often have extensive inner ear hair cell damage, with physiological functions approaching irreversible change, meaning that even early intervention may not significantly improve ultimate hearing recovery.

Studies have shown that the correlation between hypertension, diabetes, and the prognosis of SSNHL remains controversial. This study found that among patients with severe to profound SSNHL, the presence of hypertension or diabetes was not significantly associated with hearing recovery, a finding consistent with some previous studies [34], although other studies failed to support these conclusions. A prospective cohort study by Zand et al. [54] reported that patients with metabolic syndrome (especially those with comorbid hypertension or diabetes mellitus) had significantly lower hearing improvement rates, identifying hypertension and diabetes as independent risk factors for poor prognosis. Conversely, some studies have even suggested that diabetes may be a positive prognostic factor [43]. Seo et al. [55] found that after matching for age, sex, and initial hearing level, although diabetic patients had worse initial hearing thresholds, their complete recovery rate was not significantly different from that of the control group. This suggests that diabetes itself may not be a core factor directly leading to poor prognosis, but rather may be collinear with other confounding variables such as advanced age. The difference between our findings and previous studies may stem from heterogeneity in study populations: This study focused specifically on patients with severe to profound SSNHL, whereas previous studies had mostly studied the whole group of SSNHL. We speculate that the pathogenesis of severe to profound SSNHL may be related to cochlear vascular occlusion or thrombosis, resulting in broader cochlear damage [39]. The extensive hair cell damage in such cases may produce a “ceiling effect,” where the severity of initial damage directly determines prognosis and potentially masks the chronic microcirculatory effects of metabolic diseases like hypertension and diabetes. Therefore, the severity of initial hearing loss itself may be a more influential prognostic factor than hypertension and diabetes. In sensitivity analyses, the association between diabetes and poor prognosis showed instability, suggesting that conclusions about diabetes as a risk factor for poor prognosis in severe to profound SSNHL may be significantly affected by study heterogeneity. Future studies should aim to expand sample sizes, standardize definitions of hypertension and diabetes, and conduct subgroup analyses to further clarify their clinical significance.

This study also has several limitations. First, all included studies were retrospective, and there were differences in the characteristics and ethnic distribution of study populations, inevitably leading to clinical heterogeneity. Second, on the one hand, there is currently no internationally recognized gold standard for prognosis evaluation in the field of SSNHL research. The prognostic criteria adopted by different studies were not completely consistent, and the definition of “poor prognosis” varied, which may be one of the major sources of heterogeneity. On the other hand, differences in the hearing classification standards used across studies led to discrepancies in the threshold definitions for “severe” and “profound” hearing loss, and this lack of standardization increased the difficulty of comparing efficacy across studies. Third, although some studies mentioned potential prognostic factors such as auditory brainstem response and vestibular evoked myogenic potentials, due to the limited number of relevant studies, effective and reasonable meta-analyses could not be conducted. Future studies should focus on conducting large-scale, multicenter, prospective studies and establishing a standardized prognostic evaluation system to provide higher-quality evidence for the precise diagnosis and treatment of severe to profound SSNHL.

## 5. Conclusions

To our knowledge, this is the first meta-analysis to examine prognostic risk factors in patients with severe to profound SSNHL, providing evidence-based support for clinical diagnosis and treatment. This study demonstrates that the severity of initial hearing loss and the presence of vertigo are statistically significant risk factors for poor prognosis in patients with severe to profound SSNHL. Based on these findings, it is recommended that clinical evaluation of such patients prioritize assessment of the initial hearing threshold and vertigo symptoms to facilitate early identification of high-risk individuals and implement personalized intervention strategies.

## Figures and Tables

**Table 1:** Criteria of hearing recovery (according to Siegel’s criteria).

Type	Hearing Recovery
Complete recovery (CR)	Final hearing threshold < 25 dB HL
Partial recovery (PR)	Hearing gain ≥ 15 dB HL and final threshold 25–45 dB HL
Slight Improvement (SI)	Hearing gain ≥ 15 dB HL and final threshold > 45 dB HL
No improvement (NI)	Hearing gain < 15 dB HL and final threshold > 75 dB HL

**Table 2:** Characteristics of included studies in terms of population, treatment, and prognosis.

Study	Country	Design	Severe (*n*)	Profound (*n*)	Treatment	Hearing Improvement Criteria	Prognostic Factor
Askar (2023) [18]	Egypt	CS	45	22	ITS	Siegel’s criteria (≥15 dB HL hearing gain)	Severity of hearing loss
Cho (2022) [19]	Korea	CS	84	94	OS	Siegel’s criteria	Severity of hearing loss, Vertigo, Delayed treatment
Choi (2020) [20]	Korea	RC	-	103	SST/SST + ITS, salvage ITS	Siegel’s criteria (≥15 dB HL hearing gain and a final hearing better than 45 dB HL)	Gender, Vertigo, Hypertension, Diabetes
Jo (2015) [21]	Korea	CS	77	225	IM, P, MS, PE1, C, ITS	Siegel’s criteria	Severity of hearing loss
Lee (2014) [22]	Korea	CS	66	54	IM, OS, GFG, SGB	Siegel’s criteria (≥15 dB HL hearing gain)	Severity of hearing loss
Perez (2021) [23]	Brazil	RC	48	71	OS	AAO-HNSF (≥10 dB HL improvement in PTA, PTA ≤ 50 dB HL and WRS ≥ 50%)	Severity of hearing loss
Wang (2009) [24]	China	RC	88 (Specific subgroup numbers not provided)		OS, RR	Complete recovery (hearing improvement > 30 dB HL and side difference ≤ 10 dB HL) marked recovery (hearing improvement > 30 dB HL) mild recovery (10 dB HL < hearing improvement ≤ 30 dB HL) no recovery (hearing improvement ≤ 10 dB HL)	Gender, Hypertension, Diabetes, Vertigo, Tinnitus, Age
Weiss (2017) [25]	Germany	RC	198	-	PE, LS, HS	Siegel’s criteria (≥15 dB HL hearing gain)	Gender, Vertigo, Tinnitus
Wen (2014) [26]	China	RC	-	576	OS, salvage ITS	Siegel’s criteria (≥15 dB HL hearing gain)	Gender, Hypertension, Diabetes, Vertigo, Tinnitus
Yang (2023) [27]	China	CS	14	35	IV, ITS, HBO	PTA improvement > 30 dB HL	Gender, Vertigo, Delayed treatment
You (2023) [28]	China	CS	-	75	OS/IV, I, EP, CP, HBO	PTA improvement > 30 dB HL	Age, Vertigo
Zhang (2023) [29]	China	RC	61	60	SST/LST, M, GBE, B, HBO	Siegel’s criteria (≥15 dB HL hearing gain)	Severity of hearing loss
Lee (2002) [30]	Korea	CS	50	75	IP	Siegel’s criteria (≥15 dB HL hearing gain)	Severity of hearing loss
Dai (2025) [31]	China	RC	-	191	IV, ITS, B, GBE, M	Chinese Medical Association of Otolaryngology criteria	Vertigo
Zhao (2025) [32]	China	RC	-	252	Not reported	AAO-HNSF (PTA ≤ 50 dB HL and WRS ≤ 50%)	Gender, Hypertension, Diabetes, Tinnitus, Vertigo

CS retrospective case series, ITS intratympanic steroid, OS oral steroids, IM intramuscular methylprednisolone, P pentaspan, MS magnesium sulfate, PE1 prostaglandin E1, C carbogen (95% O_2_ + 5% CO_2_), GFG gingko flavone glycoside, SGB stellate ganglion block, RC retrospective cohort, RR rheomac rodex, PE pentoxifylline, LS low-dose steroids, HS high-dose steroids, IV intravenous steroids, PIS postauricular injection steroid, GBE Ginkgo biloba extract, M monosialoganglioside, HBO hyperbaric oxygen, I ibuprofen, EP enalapril, CP captopril, SST systemic steroid therapy, LST local steroid therapy, M mecobalamin, B batroxobin, IP intramuscular prednisolone.

**Table 3:** Summary of meta-analysis results on the association between 8 risk factors and prognosis.

Risk Factor	Studies	Sample Size	Heterogeneity		Meta-Analysis Model	OR (95% CI)	Z	*p*
			**I^2^ (%)**	**P_h_**				
Severity of hearing loss (profound vs. severe)	8	1120	0	0.62	Fixed-effect model	4.68 (3.57, 6.13)	11.22	<0.001
Gender (male vs. female)	6	1266	1	0.41	Fixed-effect model	0.80 (0.63,1.02)	1.78	0.08
Age (>60 vs. ≤60 years)	2	163	0	0.42	Fixed-effect model	0.94 (0.42, 2.11)	0.15	0.88
Tinnitus (presence vs. absence)	3	838	0	0.66	Fixed-effect model	0.84 (0.61, 1.15)	1.08	0.28
Delayed treatment (>7 vs. ≤7 days)	2	191	0	0.9	Fixed-effect model	1.08 (0.26, 4.51)	0.11	0.91
Vertigo (presence vs. absence)	9	1014	70	0.0009	Random-effects model	1.95 (1.28, 2.98)	3.10	0.002
Hypertension (presence vs. absence)	4	434	67	0.03	Random-effects model	1.02 (0.49, 2.14)	0.06	0.95
Diabetes (presence vs. absence)	4	452	55	0.09	Random-effects model	1.15 (0.62, 2.11)	0.44	0.66

## Data Availability

All data generated or analyzed during this study are included in this article/Supplementary material, further inquiries can be directed to the corresponding authors.

## Supplementary Materials

The following supporting information can be downloaded at: https://www.mdpi.com/article/10.3390/diagnostics15212770/s1, Supplementary File S1: PRISMA checklist; Supplementary File S2: Database Search Strategies; Figure S1: Funnel plots showing publication bias for subgroups of severity of hearing loss (a), vertigo (b), and gender (c); Table S1: Assessment of included cohort studies using the Newcastle-Ottawa Scale (NOS); Table S2: Assessment of included case series studies using the Joanna Briggs Institute (JBI) critical appraisal tool; Table S3: Definition of SSNHL and criteria for the grading of hearing loss
