# Supplementary material for: Analysis of the Reasons for Poor Prognosis in Severe to Profound Sudden Sensorineural Hearing Loss: A Systematic Review and Meta-Analysis
Source: Diagnostics (Basel). 2025 Oct 31;15(21):2770. doi: 10.3390/diagnostics15212770 (PMC12607817; doi:10.3390/diagnostics15212770)
Supplement: Supplementary file 1 [file diagnostics-15-02770-s001.zip › diagnostics-3881098-supplementary.pdf]

Supplementary material

S2. Database Search Strategies

PubMed

Pubmed		Total
#1	("Hearing Loss, Sudden"[Mesh]) OR (((((((sudden hearing loss[Title/Abstract]) OR (deafness, sudden[Title/Abstract])) OR (sudden deafness[Title/Abstract])) OR (idiopathic sudden sensorineural hearing loss[Title/Abstract])) OR (sudden sensorineural hearing loss[Title/Abstract])) OR (SSNHL[Title/Abstract])) OR (SNHL[Title/Abstract]))	7,746
#2	(((severe to profound[Title/Abstract]) OR (severe-to-profound[Title/Abstract])) OR (severe[Title/Abstract])) OR (profound[Title/Abstract])	1,481,531
#3	("Prognosis"[Mesh]) OR ((((((((((prognoses[Title/Abstract]) OR (prognosticfactors[Title/Abstract])) OR (prognostic factor[Title/Abstract])) OR (factor, prognostic[Title/Abstract])) OR (factors, prognostic[Title/Abstract])) OR (age[Title/Abstract])) OR (vertigo[Title/Abstract])) OR (tinnitus[Title/Abstract])) OR (hypertension[Title/Abstract])) OR (diabetes[Title/Abstract]))	5,763,457
#4	#3 AND #2 AND #1	794

Web of Science

Web of Science		Total
#1	TS=(hearing Loss, sudden OR sudden hearing loss OR deafness, sudden OR sudden deafness OR idiopathic sudden sensorineural hearing loss OR sudden sensorineural hearing loss OR SSNHL OR SNHL)	11,009
#2	TS=(severe to profound OR severe-to-profound OR severe OR profound)	2,431,362
#3	TS=(severe to profound OR severe-to-profound OR severe OR profound)	2,431,362
#4	#3 AND #2 AND #1	1,401

Embase

Embase		Total
#1	hearing Loss, sudden '/exp OR 'SNHL':ab,ti OR 'SSNHL':ab,ti OR 'sudden sensorineural hearing loss':ab,ti OR 'idiopathic sudden sensorineural hearing loss':ab,ti OR 'sudden deafness':ab,ti OR 'deafness, sudden':ab,ti OR 'sudden hearing loss':ab,ti OR 'factors, prognostic':ab,ti	10,035
#2	'prognosis'/exp OR 'factors, prognostic':ab,ti OR 'factor, prognostic':ab,ti OR 'prognostic factor':ab,ti OR 'prognosticfactors':ab,ti OR 'prognoses':ab,ti OR B'diabetes':ab,ti OR 'hypertension':ab,ti OR 'tinnitus':ab,ti OR 'vertigo':ab,ti OR 'age':ab,ti	7,342,475
#3	'profound':ab,ti OR 'severe':ab,ti OR 'severe-to-profound':ab,ti OR	2,193,155

	'severe to profound':ab,ti	
#4	#3 AND #2 AND #1	960

The Cochrane Library

The Cochrane Library		Total
#1	MeSH descriptor: [Hearing Loss, Sudden] explode all trees	249
#2	(sudden hearing loss): ti,ab,kw OR (deafness, sudden): ti,ab,kw OR (sudden deafness): ti,ab,kw OR (idiopathic sudden sensorineural hearing loss): ti,ab,kw OR (sudden sensorineural hearing loss): ti,ab,kw OR (SSNHL): ti,ab,kw OR (SNHL)	538
#3	#2 AND #1	787
#4	MeSH descriptor: [prognosis] explode all trees	230144
#5	(prognoses): ti,ab,kw OR (prognosticfactors): ti,ab,kw OR (prognostic factor): ti,ab,kw OR (factor, prognostic): ti,ab,kw OR (factors, prognostic): ti,ab,kw OR (age): ti,ab,kw OR (vertigo): ti,ab,kw OR (tinnitus): ti,ab,kw OR (hypertension): ti,ab,kw OR (diabetes)	936265
#6	#4 AND #5	1166409
#7	(severe to profound): ti,ab,kw OR (severe-to-profound): ti,ab,kw OR (severe): ti,ab,kw OR (profound): ti,ab,kw	332371
#8	#3 AND #6 AND #7	127

Publication biases

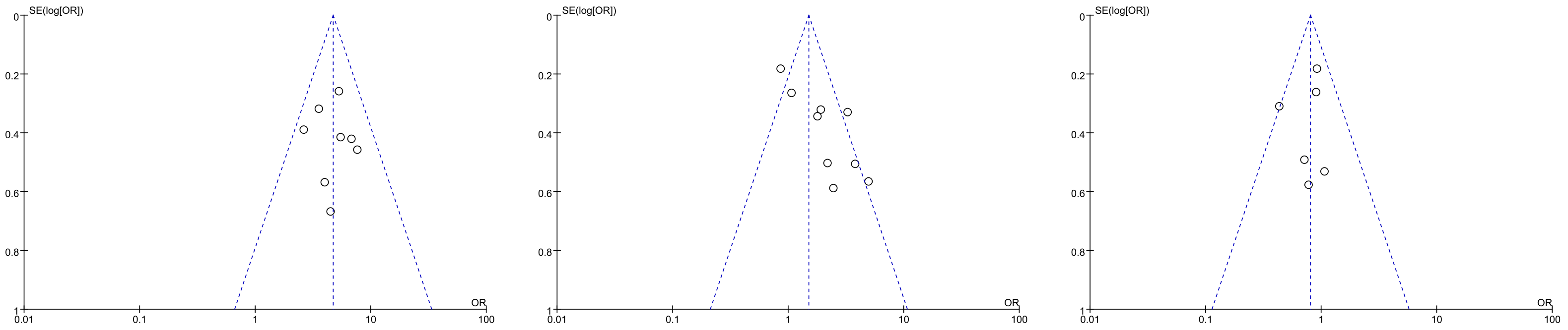

(a) (b) (c)

Figure S1. Funnel plots showing publication bias for subgroups of severity of hearing loss (a), vertigo (b), and gender (c)

Quality assessment

Table S1. Assessment of included cohort studies using the Newcastle-Ottawa Scale (NOS)

Author	Selection	Comparability	Outcome	Total score
Choi (2020)	4/4	2/2	3/3	9
Perez (2021)	4/4	2/2	2/3	8
Wang (2009)	4/4	2/2	2/3	8
Weiss (2017)	4/4	1/2	2/3	7
Wen (2014)	4/4	1/2	2/3	7
Zhang (2023)	4/4	2/2	2/3	8
Dai (2025)	4/4	2/2	2/3	8
Zhao (2025)	4/4	2/2	2/3	8

Table S2. Assessment of included case series studies using the Joanna Briggs Institute (JBI) critical appraisal tool

Author	Were there clear criteria for inclusion in the case series?	Was the condition measured in a standard, reliable way for all participants included in the case series?	Were valid methods used for identification of the condition for all participants included in the case series?	Did the case series have consecutive inclusion of participants?	Did the case series have complete inclusion of participants?	Was there clear reporting of the demographics of the participants in the study?	Was there clear reporting of clinical information of the participants?	Were the outcomes or follow up results of cases clearly reported?	Was there clear reporting of the presenting site(s)/clinic(s) demographic information?	Was statistical analysis appropriate?	Points
Askar (2023)	+	+	+	+	**-**	+	+	+	+	+	**9/10**
Cho (2022)	+	+	+	+	**U**	+	+	+	+	+	**9/9**
Jo (2015)	+	+	+	+	**U**	+	+	+	+	+	**9/9**
Lee (2014)	+	+	+	+	**U**	+	+	+	+	+	**9/9**
Yang (2023)	+	+	+	+	**U**	+	+	+	+	+	**9/9**
You (2023)	+	+	+	+	**U**	+	+	+	+	+	**9/9**
Lee	+	+	+	+	**U**	+	+	+	+	+	**9/9**

2002)

Abbreviations: “+” for “Yes”, “**-“** for “No”, “U”-“Unclear”

Characteristics table

Table S3. Definition of SSNHL and criteria for the grading of hearing loss

Study	Definition of SSNHL	Criteria for the grading of hearing loss
Askar (2023)	Hearing loss of more than 30 dB HL in the thresholds for at least three consecutive frequencies within 3 days	The Research Committee of the Ministry of Health and Welfare in Japan: severe: 60 dB HL ≤PTA <90 dB HL; profound: PTA ≥90 dB HL (500, 1k, 2k, and 4 kHz)
Cho (2022)	Hearing loss of more than 30 dB HL in the thresholds for at least three consecutive frequencies within 3 days	Severe:70 dB HL < PTA ≤90 dB HL; profound: PTA ≥91 dB HL (500, 1k, 2k, and 4 kHz)
Choi (2020)	Profound SSNHL: unexplained unilateral sudden SSNHL≥90 dB HL hearing loss at least four consecutive frequencies occurring within 3 days	Profound: PTA≥90 dB HL (500, 1k, 2k, and 4k Hz)
Lee (2014)	Hearing loss of more than 30 dB HL in the thresholds for at least three consecutive frequencies within 3 days	Severe:70 dB HL < PTA ≤90 dB HL; profound: PTA ≥91 dB HL (500, 1k, 2k, and 3 kHz)
Perez (2021)	Hearing loss of more than 30 dB HL in the thresholds for at least three consecutive frequencies within 3 days	Severe:70 dB HL < PTA ≤90 dB HL; profound: PTA ≥91 dB HL (500, 1k, 2k, and 3 kHz)
Wang (2009)	Hearing loss of more than 30 dB HL in the thresholds for at least three consecutive frequencies within 3 days	Severe:70 dB HL < PTA ≤90 dB HL; profound: PTA ≥91 dB HL (250, 500, 1k, 2k, and 4 kHz)
Jo (2015)	Hearing loss of more than 30 dB HL in the thresholds for at least three consecutive frequencies within 3 days	Severe to profound: PTA≥70 dB HL (500, 1k, 2k, and 4 kHz)
Weiss (2017)	Severe SSNHL: an acute, unilateral SSNHL ≥60 dB HL in at least 4 frequencies between 125 and 8000 Hz compared to the healthy ear	Severe to profound: PTA≥60 dB HL (250, 500, 1k, 2k, 3k and 4 kHz)
Wen (2014)	Hearing loss of more than 30 dB HL in the thresholds for at least three consecutive frequencies within 3 days	Profound: PTA≥90 dB HL (500, 1k, 2k and 4k Hz)
Yang (2023)	Hearing loss of more than 30 dB HL in the thresholds for at least three consecutive frequencies within 3 days	According to the standard of the World Health Organization (2021), severe: 65 dB HL ≤ PTA <80 dB HL; profound: ≥80 dB HL (500, 1k, 2k, and 4 kHz)

You (2023)	Hearing loss of more than 20 dB HL in the thresholds for at least two consecutive frequencies within 3 days	Total SSNHL: Hearing loss in each frequency band: 250 ~ 8000 Hz (250, 500, 1k, 2k, 3k, 4k, 8kHz), and the average hearing threshold is ≥81 dB HL
Zhang (2023)	Hearing loss of more than 30 dB HL in the thresholds for at least three consecutive frequencies within 3 days	According to the standard of the World Health Organization (1997), severe: 61 dB HL≤ PTA ≤80 dB HL; profound: ≥81 dB HL (500, 1k, 2k, and 4 kHz)
Lee (2002)	Hearing loss of more than 30 dB HL in the thresholds for at least three consecutive frequencies within 3 days	Severe:70 dB HL < PTA ≤90 dB HL; profound: PTA ≥91 dB HL (500, 1k, 2k, and 4 kHz)
Dai (2025)	Hearing loss of more than 20 dB HL in the thresholds for at least two consecutive frequencies within 3 days	Profound: PTA > 80 dB HL (500, 1k, 2k and 4k Hz)
Zhao (2025)	Hearing loss of more than 30 dB HL in the thresholds for at least three consecutive frequencies within 3 days	Profound: PTA > 80 dB HL (500, 1k, 2k and 4k Hz)
